# Appreciation of literature by the anaesthetist: A comparison of citations, downloads and Altmetric Attention Score

**DOI:** 10.1111/aas.13575

**Published:** 2020-03-16

**Authors:** Jasper M. Kampman, Jeroen Hermanides, Pascal R. Q. Boere, Markus W. Hollmann

**Affiliations:** ^1^ Department of Anaesthesiology Amsterdam University Medical Centers University of Amsterdam Amsterdam The Netherlands

## Abstract

**Background:**

Different metrics exist to evaluate the impact of a paper. Traditionally, scientific citations are leading, but nowadays new, internet‐based, metrics like downloads or Altmetric Attention Score receive increasing attention. We hypothesised a gap between these metrics, reflected by a divergence between scientific and clinical appreciation of anaesthesia literature.

**Methods:**

We collected the top 100 most cited and the top 100 most downloaded articles in *Acta Anaesthesiologica Scandinavica (AAS)* and *Anesthesia & Analgesia (A&A)* published between 2014 and 2018. We analysed the relationship between the average number of citations per year, downloads per year and Altmetric Attention Score.

**Results:**

For both *AAS* and *A&A*, a significant correlation between the 100 most cited articles and their downloads (*r* = .573 and .603, respectively, *P* < .001) was found. However, only a poor correlation with Altmetric Attention Score was determined. For the 100 most downloaded articles, download frequency did not correlate with their number of citations (*r* = .035 and .139 respectively), but did correlate significantly with the Altmetric Attention Score (*r* = .458 and .354, *P* < .001).

**Conclusion:**

Highly cited articles are downloaded more frequently. The most downloaded articles, however, did not receive more citations. In contrast to the most cited articles, more frequently downloaded papers had a higher Altmetric Attention Score. Thus, a ‘trending’ anaesthesia paper is not a prerequisite for scientific appreciation, reflecting a gap between clinical and scientific appreciation of literature.

**Editorial Comment**

This article tells us that there seem to be a gap between scientific and the clinical appreciation of the literature, at least as judged by the relationship between citations, downloading and Altmetric Attention Scores. The rate of downloading and the Altmetric score probably reflect more clinical than scientific interest.

## 1 INTRODUCTION

The traditional way of ranking individual scientists is, among others, based on the number of citations their papers receive and the impact factor (IF) of the journals their articles are published in. In the internet era, new metrics to measure the impact of a paper, such as the number of downloads or Altmetric Attention Score, are available. Those can be used to measure the impact of publications on both the clinical and non‐clinical community. It quantifies the influence and attention an article receives by looking at social media, Wikipedia citations, reference managers, blogs, media coverage and public policy documents.^1^ In recent years, many journals have reduced or abandoned their printed issues. As a result, researchers and other interested readers have to download an article before being able to read, or cite, it. This makes the number of downloads an increasingly accurate metric for the interest an article arouses with its readership.

In this study, we examined how literature is appreciated by anaesthetists by studying the relationship between the number of citations, the number of downloads and the Altmetric Attention Score of popular anaesthesia papers. We hypothesise a gap between these metrics, reflecting a divergence between scientific and clinical appreciation of anaesthesia literature.

## 2 MATERIALS AND METHODS

### 2.1 Data collection

We reached out to two prominent anaesthesia journals, one from the United States and one from Europe, asking for their 100 most cited and most downloaded articles published between 2014 and 2018. We received data from *Wiley Online Library Denmark* illustrating the most cited and most downloaded papers from *Acta Anaesthesiologica Scandinavica*, along with their corresponding number of downloads and citations. From *Wolters Kluwer USA*, we received the same data for *Anesthesia & Analgesia*. The download frequency, an approximation of how often the paper is read, is the combined number of PDF downloads and article views online (HTML or PDF). For both citation and download analyses, we calculated averages per year since publication to compensate for the shorter time more recent articles have had to accumulate their numbers. On 24 January 2020, we collected the Altmetric Attention Scores, provided by *Altmetric.com*, from the journal websites. The complete lists of articles, citations, downloads and Altmetrics are available in the Supporting Information.

### 2.2 Statistical approach

We suspected that each list might contain one or two significant outliers, papers that received such a high number of citations or downloads that this could influence the analyses. To detect significant outliers, we calculated centred leverage values and standardised residuals for each paper in our lists. The Supporting information contain a detailed description of this process. To test the relationship between the different metrics, we used Spearman's rank correlation coefficient. Our data were analysed using the Statistical Package for the Social Sciences (SPSS, version 25).

## 3 RESULTS

### 3.1 Acta anaesthesiologica scandinavica

The top 100 most cited and 100 most downloaded from *AAS* contain a total of 146 different papers, which means that 54 papers appear in both lists. For the most cited list, the median number of citations per year is 4.2 (IQR 3.5‐5.8) and the median number of downloads per year is 212 (137‐373). For the most downloaded papers, this is 3.5 (1.6‐5.7) and 373 (274‐646) respectively.

**FIGURE 1:**
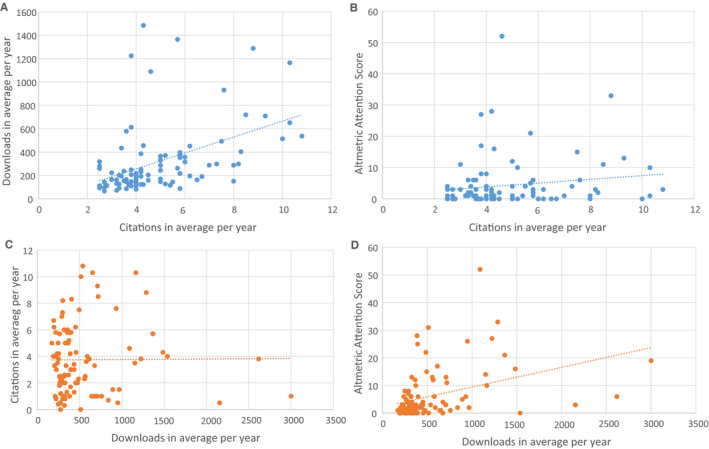
*Acta Anaesthesiologica Scandinavica*. Above: most cited articles, relationship between citations and downloads (A, *r* = .573) and Altmetric Attention Score (B, *r* = .182). Below: most downloaded articles, relationship between downloads and citations (C, *r* = .035) and Altmetric Attention Score (D, *r* = .458) [Colour figure can be viewed at wileyonlinelibrary.com]

We detected three outliers within the 100 most cited articles and two outliers within the 100 most downloaded articles. These outliers were excluded from the Spearman's test for correlation. Figure 1 shows the relationships between citation count, number of downloads and Altmetric Attention Score for both top 100's. In Figure 1A, the relationship between the most cited papers and their number of downloads is presented. The correlation coefficient (*r*) is .573, with *P* < .001. Analysis of the relation between citations and Altmetric Attention Score (Figure 1B) revealed an *r* = .182 (*P* = .074). For the most downloaded papers, Figure 1C represents the relationship between downloads and citation count, with *r* = .035 (*P* = .734). Figure 1D illustrates the correlation between downloads and Altmetric Attention Score, with *r* = .458 (*P* < .001).

When looking at the relationship between date of publication and Altmetric Attention Score for all 146 papers, a moderate correlation was found, indicating that more recent papers have higher Altmetric Attention Scores (Figure 3A, *r* = .170, *P* = .04). Figure 3C shows the correlation between publication date and downloads, which gives *r* = .185, *P* = .025.

### 3.2 Anesthesia & analgesia

The most cited and most downloaded from *A&A* lists consist of 167 individual articles, meaning 33 appear in both top 100's. The median number of citations per year in the most cited list is 10 (IQR 7.4‐13.2) and the median number of downloads is 453 (299‐704). For the most downloaded list, this is, respectively, 6.4 (3‐12.3) and 1147 (714‐1849).

**FIGURE 2:**
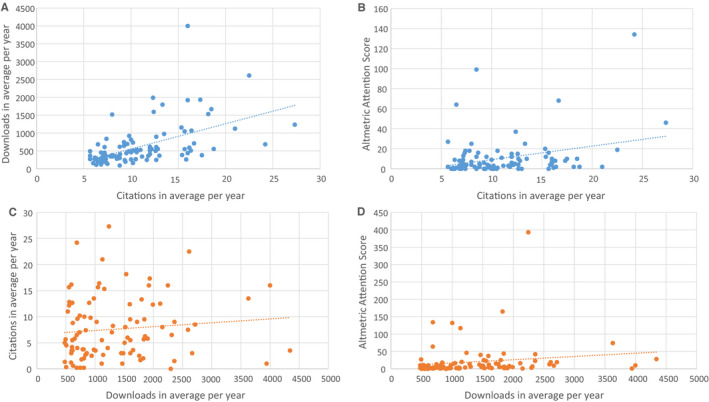
*Anesthesia & Analgesia*. Above: most cited articles, relationship between citations and downloads (A, *r* = .603) and Altmetric Attention Score (B, *r* = .210). Below: most downloaded articles, relationship between downloads and citations (C, *r* = .139) and Altmetric Attention Score (D, *r* = .354) [Colour figure can be viewed at wileyonlinelibrary.com]

Two outliers were detected in the most cited list and four outliers in the most downloaded list. These were excluded in the correlation analyses, which are shown in Figure 2. The most cited articles show a strong correlation with their number of downloads with *r* = .603 (*P* < .001) and a poor correlation with the Altmetric Attention Score (*r* = .210, *P* = .038), respectively, as shown in Figure 2A and B. The most downloaded articles show no correlation with their number of citations (*r* = .139, *P* = .176, Figure 2C), but a relatively strong correlation with Altmetric Attention Score, *r* = .354 (*P* < .001, Figure 2D).

**FIGURE 3:**
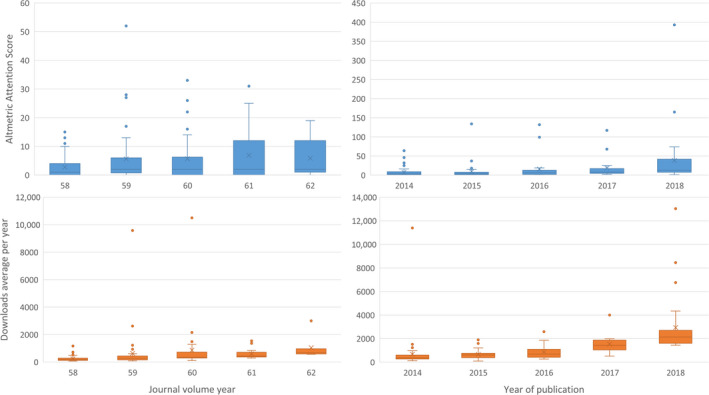
Correlation with year of publication. Above: Altmetric Attention Score in *Acta Anaesthesiologica Scandinavica* (A, *r* = .170) and *Anesthesia & Analgesia* (B, *r* = .269). Below: downloads in *Acta Anaesthesiologica Scandinavica* (C, *r* = .185) and *Anesthesia & Analgesia* (D, *r* = .458) [Colour figure can be viewed at wileyonlinelibrary.com]

Figure 3B illustrates the publishing year of all 167 individual articles in our top 100's in relation to their Altmetric Attention Scores, *r* = .269 (*P* < .001), indicating higher scores for more recent papers. The correlation between year of publication and downloads, Figure 3D, revealed an *r* = .458 (*P* < .001).

## 4 DISCUSSION

This is the first study comparing between citations, downloads and Altmetric Attention Score within the field of anaesthesia. A large number of best performing papers published between 2014 and 2018 in *Acta Anaesthesiologica Scandinavica* and *Anesthesia & Analgesia* was analysed.

### 4.1 Main results and conclusions

For both journals, we see a strong correlation between citations and downloads for the top 100 most cited articles. Thus, frequently cited articles are read more often, most likely indicating scientific interest in particular. Contrarily, the 100 most downloaded articles, which received about twice as many reads as the most cited papers, show no correlation between the number of downloads and citation count. A possible explanation for the absence of correlation could be that some highly frequently downloaded papers, like new guidelines, generate a lot of public attention and are heavily read, even though the interest of academia is limited. Another explanation might be found in the relatively low research output of anaesthesia compared to other specialties.^2^ Anaesthesia is in second to last place on a list of 25 medical specialties in the United States when looking at public research funding, an indicator of research productivity.^3^ Our results show a divergence in academic and clinical literary interest, which might be illustrative for the underlying reason of our low output, the insufficient integration of research and daily clinical practice.

Secondly, we found that the 100 most downloaded articles revealed a strong correlation with the Altmetric Attention Score, while the 100 most cited articles did not. The Altmetric Attention Score is generated quickly after publication, making it a potential predictor of more slowly acquired metrics, such as citations or downloads. Our data suggest that Altmetrics might be a good indicator of clinical interest and frequency of reading, but does not predict scientific interest and later citations.

Furthermore, we found a significant correlation between the publication date and the Altmetric Attention Score. This illustrates ‘recent bias’, whereby metrics are influenced by the duration of a paper present in literature.^4^ Therefore, it seems to be an unfair measure for comparing articles published in different years. However, once the use of Altmetric input variables, like social media, stabilises, the usefulness of Altmetric Attention Score as a way to evaluate papers might increase.^5^

We found an even stronger correlation between the publication date and the frequency an article is read, reflecting the same recent bias we found for Altmetrics. For *Acta Anaesthesiologica Scandinavica*, this might be explained by their opt‐out policy introduced in 2016, where subscribers have the option to cancel their printed version, receiving only online access. As far as we know, *Anesthesia & Analgesia* does not offer such a service.

### 4.2 Comparison with previous studies

This is the first study to compare citations, downloads and Altmetric Attention Scores in anaesthesia literature. Outside of anaesthesia, several studies have looked at the relationship between traditional citation‐based metrics and Altmetrics.^6^, ^7^, ^8^ Similar to our results, these studies found a poor correlation between citations and social media Altmetrics. Only early reference manager reads showed a significant correlation with later citation count.^8^

Prior studies have commented on existing metrics and the tendency in the scientific community to look at those as the single measure of scientific quality. In particular, citation‐based metrics have received criticism. Even Hirsch himself, when introducing his *h‐index*, wrote that it could only provide an indication of scientific value.^9^ There is no ‘magic tool to measure the unmeasurable—the quality of research’ as Kreiner stated in his article about the *Hirsch index*.^10^ Journal IF also seems insufficient to judge the quality of an individual research, given the lack of correlation between IF and individual article citations.^11^ Gisvold argued that it is not research quality that determines the number of citations, but rather the medical specialty one publishes in (field effect) and whether one publishes on a popular topic that receives much scientific attention, also known as the band wagon effect.^12^

Despite being fairly new, many journal websites display an Altmetric Attention Score with their articles and the Altmetric Attention Score has also received attention in literature. Contrary to citations, the input variables for the Altmetric Attention Score are generated quickly after publication, which facilitates a fast and easy evaluation. Altmetric Attention Score is an excellent indicator of public interest, and although the importance of public engagement in research should not be disregarded, it might fall short in predicting scientific quality. A study assessing the topics of the 100 highest scoring Altmetric articles revealed medical and health sciences being the most popular theme, with 36 papers in the top 100. However, the interest was limited to topics directly relevant to the public, such as diet and exercise.^5^

### 4.3 Limitations and strengths

The different metrics we studied are dynamic and might continue to grow for decades, which is in particular true for citations. Consequently, our data might differ if we repeat this study a year from now. In an attempt to compensate for timing bias, we used the average number of citations and downloads per year since publication.

A strength of our study is that we analysed data from two different anaesthesia journals, a European journal and an American journal. The fact that both analyses yielded nearly identical results strengthens our observations.

### 4.4 Conclusion

Highly cited articles are downloaded more frequently and, hopefully, read. Contrarily, the highly downloaded articles revealed no correlation between download frequency and citations count, showing that most of these downloads do not necessarily indicate scientific interest. There was, however, a strong correlation between downloads and Altmetric Attention Score. Thus, the number of downloads and Altmetrics both reflect a more short‐term interest in a paper. However, such a ‘trending’ anaesthesia paper is not a prerequisite for scientific appreciation, supporting our hypothesis of a gap in the way scientific literature is appreciated by researchers and clinicians in the field of anaesthesia.

## CONFLICT OF INTEREST

The authors certify that there is no conflict of interest with any financial organisation regarding the material discussed in the article.

## Supporting information

Supplementary Material
