# Supplementary material for: Appreciation of literature by the anaesthetist: A comparison of citations, downloads and Altmetric Attention Score
Source: Acta Anaesthesiol Scand. 2020 Mar 16;64(6):823–8. doi: 10.1111/aas.13575 (PMC7317916; doi:10.1111/aas.13575)
Supplement: Supplementary file 1 — Supplementary Material [file AAS-64-823-s001.docx]

**Supplementary data**

**Investigating outliers**

Looking at the scatterplots of the 100 most cited and 100 most downloaded a few obvious outliers stand out that might influence the correlation coefficient. For each of the papers in both lists we calculated centred leverage values and standardised residuals. We produced a scatter plot of the centred leverage values and the standardised residuals. We used a residuals cut off value of 3 for extreme outliers and for the leverage cut off a value of 3*(k+1)/n where k is the number of independent variables and n the number of articles. In this case the number of citations was the only independent variable and our lists contain 100 articles, so the leverage cut off was 3*(1+1)/100 = 0,06.

**Most cited *Acta Anaesthesiologica Scandinavica***

Scatterplot of standardised residuals and leverage. Three papers reached outside of the cut off lines (0,06 on x-axis, -3 and 3 on y-axis), these are numbers 1, 2 and 68 of the top 100 list.


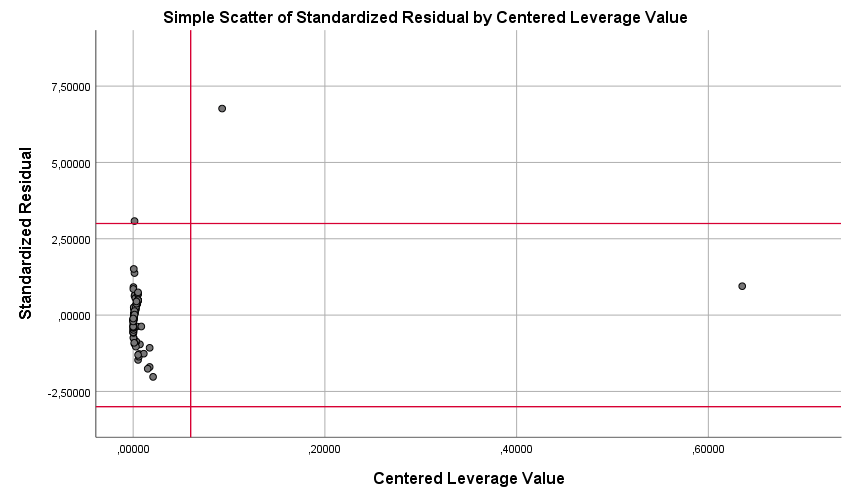


**Most downloaded *Acta Anaesthesiologica Scandinavica***

Scatterplot of standardised residuals and leverage. Two papers reached outside of the cut off lines (0,06 on x-axis, -3 and 3 on y-axis), these are numbers 1 and 2 of the top 100 list.


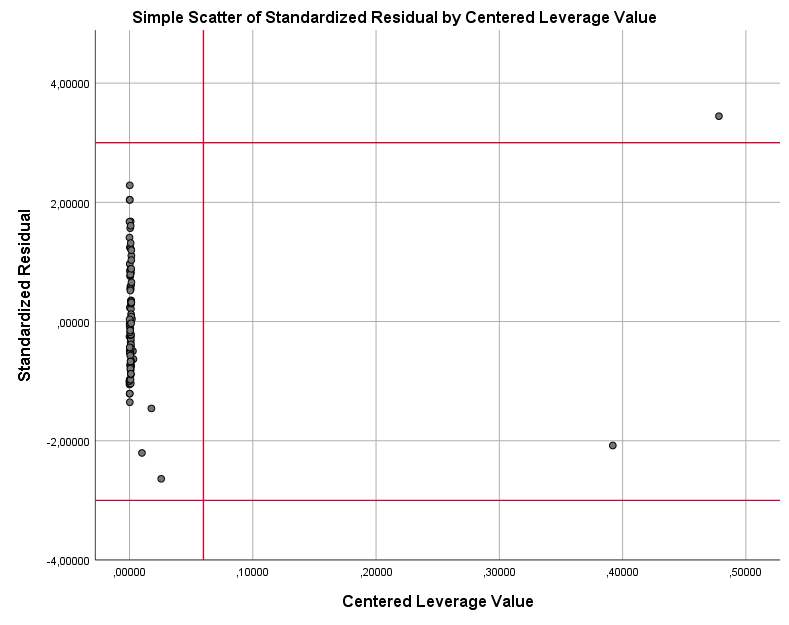


**Most cited *Anesthesia & Analgesia***

Scatterplot of standardised residuals and leverage. Two papers reached outside of the cut off lines (0,06 on x-axis, -3 and 3 on y-axis), these are numbers 1 and 19 of the top 100 list.


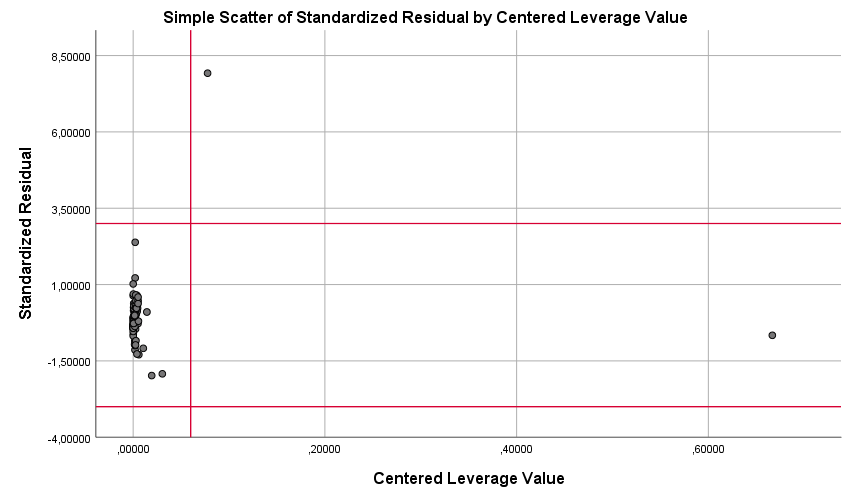


**Most downloaded *Anesthesia & Analgesia***

Scatterplot of standardised residuals and leverage. Four papers reached outside of the cut off lines (0,06 on x-axis, -3 and 3 on y-axis), these are numbers 1, 2, 3 and 4 of the top 100 list.


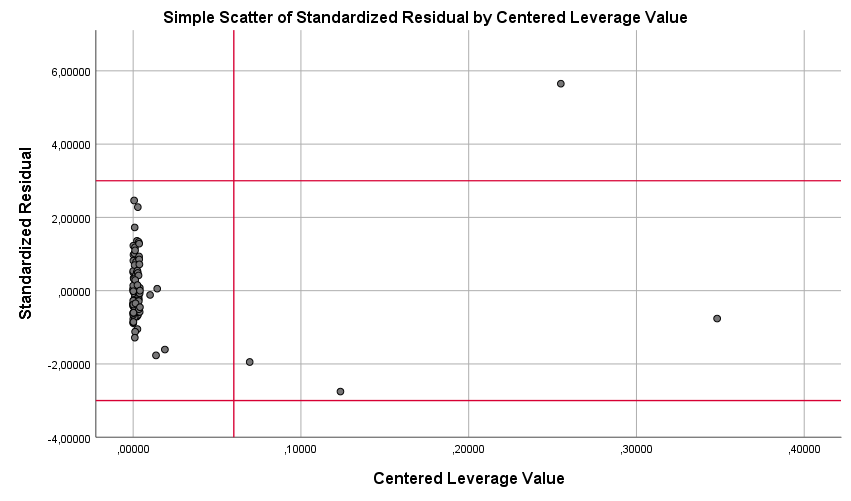


**Tables top 100 most cited and most downloaded**

**Supplementary Table 1: Top 100 most cited papers in *Acta Anaesthesiologica Scandinavica* 2014-2018 sorted by total number of citations since publication.**

|  | Paper | Journal volume | Citations per year | Downloads per year | Altmetric Attention Score |
| --- | --- | --- | --- | --- | --- |
| 1 | Enhanced Recovery After Surgery (ERAS) for gastrointestinal surgery, part 2: consensus statement for anaesthesia practice | 60 | 36,0 | 10506,3 | 7 |
| 2 | Enhanced Recovery After Surgery (ERAS) for gastrointestinal surgery, part 1: pathophysiological considerations | 59 | 17,0 | 9582,8 | 3 |
| 3 | Adverse effects of perioperative paracetamol, NSAIDs, glucocorticoids, gabapentinoids and their combinations: a topical review | 58 | 10,8 | 536,8 | 3 |
| 4 | Post-operative analgesic effects of paracetamol, NSAIDs, glucocorticoids, gabapentinoids and their combinations: a topical review | 58 | 10,3 | 652,0 | 1 |
| 5 | Clinical guidelines on central venous catheterisation | 58 | 10,3 | 1165,2 | 10 |
| 6 | The effects of safety checklists in medicine: a systematic review | 58 | 8,5 | 718,8 | 11 |
| 7 | Definition, prevalence, and outcome of feeding intolerance in intensive care: a systematic review and meta-analysis | 58 | 8,3 | 403,3 | 2 |
| 8 | Neuromuscular blockade for optimising surgical conditions during abdominal and gynaecological surgery: a systematic review | 59 | 10,0 | 514,8 | 0 |
| 9 | Dexmedetomidine provides neuroprotection: impact on ketamine-induced neuroapoptosis in the developing rat brain | 58 | 8,0 | 151,7 | 1 |
| 10 | A systematic review and meta-analysis of ketamine for the prevention of persistent post-surgical pain | 58 | 7,5 | 493,3 | 15 |
| 11 | Stress ulcer prophylaxis in the intensive care unit: an international survey of 97 units in 11 countries | 59 | 8,2 | 299,0 | 3 |
| 12 | Mortality in elderly ICU patients: a cohort study | 58 | 6,7 | 191,0 | 0 |
| 13 | Complex regional pain syndrome type I: a comprehensive review | 59 | 7,6 | 931,6 | 6 |
| 14 | Ultrasonography- guided radial artery catheterization is superior compared with the traditional palpation technique | 58 | 6,2 | 195,2 | 0 |
| 15 | Gabapentin for post-operative pain management - a systematic review with meta-analyses and trial sequential analyses | 60 | 9,3 | 709,8 | 13 |
| 16 | Dexmedetomidine for preventing sevoflurane-related emergence agitation in children: a meta-analysis of randomized controlled trials | 58 | 6,0 | 315,8 | 0 |
| 17 | Myocardial protection by remote ischaemic pre- conditioning is abolished in sulphonylurea- treated diabetics undergoing coronary revascularisation | 58 | 5,8 | 88,2 | 2 |
| 18 | The spread of injectate during saphenous nerve block at the adductor canal: a cadaver study | 59 | 7,0 | 288,2 | 1 |
| 19 | Scandinavian SSAI clinical practice guideline on pre-hospital airway management | 60 | 8,8 | 1288,0 | 33 |
| 20 | Fibrinogen concentrate for bleeding - a systematic review | 58 | 5,7 | 262,5 | 5 |
| 21 | Perioperative epidural analgesia reduces cancer recurrence after gastro-oesophageal surgery | 58 | 5,5 | 144,2 | 4 |
| 22 | Efficacy of simulation-based trauma team training of non-technical skills. A systematic review | 58 | 5,2 | 372,0 | 10 |
| 23 | Delayed quadriceps weakness after continuous adductor canal block for total knee arthroplasty: a case report | 58 | 5,2 | 128,3 | 0 |
| 24 | Total intravenous general anaesthesia vs. spinal anaesthesia for total hip arthroplasty: a randomised, controlled trial | 59 | 6,2 | 451,6 | 3 |
| 25 | Indocyanine green plasma disappearance rate as dynamic liver function test in critically ill patients | 58 | 5,0 | 170,5 | 1 |
| 26 | Assessing Nurse Anaesthetists' Non-Technical Skills in the operating room | 58 | 5,0 | 244,0 | 1 |
| 27 | Optimising abdominal space with deep neuromuscular blockade in gynaecologic laparoscopy - a randomised, blinded crossover study | 59 | 5,8 | 217,0 | 1 |
| 28 | Chronic pre-operative opioid use and acute pain after fast-track total knee arthroplasty | 60 | 7,3 | 297,5 | 4 |
| 29 | Monitoring hypnotic effect and nociception with two EEG-derived indices, qCON and qNOX, during general anaesthesia | 58 | 4,5 | 158,7 | 0 |
| 30 | Real-time ventilation and perfusion distributions by electrical impedance tomography during one-lung ventilation with capnothorax | 59 | 5,4 | 115,8 | 1 |
| 31 | Circulating glycosaminoglycan species in septic shock | 58 | 4,3 | 122,5 | 1 |
| 32 | Chronic non-cancer pain and the epidemic prescription of opioids in the Danish population: trends from 2000 to 2013 | 60 | 6,5 | 162,3 | 0 |
| 33 | Ultrasound-guided block of the suprascapular nerve - a volunteer study of a new proximal approach | 58 | 4,2 | 242,2 | 1 |
| 34 | Norwegian survival prediction model in trauma: modelling effects of anatomic injury, acute physiology, age, and co-morbidity | 58 | 4,2 | 231,8 | 3 |
| 35 | Decisions to withhold or withdraw life-sustaining treatment in a Norwegian intensive care unit | 58 | 4,2 | 123,5 | 1 |
| 36 | Continuous positive airway pressure/pressure support pre-oxygenation of morbidly obese patients | 58 | 4,0 | 218,3 | 1 |
| 37 | Medical emergency team activation: performance of conventional dichotomised criteria versus national early warning score | 58 | 4,0 | 193,8 | 0 |
| 38 | Analgesia Nociception Index (ANI) to predict intraoperative haemodynamic changes: results of a pilot investigation | 58 | 4,0 | 186,5 | 1 |
| 39 | Is deep neuromuscular block beneficial in laparoscopic surgery? No, probably not | 60 | 6,0 | 357,8 | 0 |
| 40 | Anaesthetic hypersensitivity reactions in France between 2011 and 2012: the 10th GERAP epidemiologic survey | 61 | 8,0 | 288,0 | 0 |
| 41 | Major complications of epidural anesthesia: a prospective study of 5083 cases at a single hospital | 58 | 3,8 | 249,2 | 8 |
| 42 | Thromboelastometry as a supplementary tool for evaluation of hemostasis in severe sepsis and septic shock | 58 | 3,8 | 147,7 | 0 |
| 43 | Crew Resource Management in the Intensive Care Unit: a prospective 3-year cohort study | 59 | 4,6 | 1089,4 | 52 |
| 44 | Comparing efficacy and safety of fibrinogen concentrate to cryoprecipitate in bleeding patients: a systematic review | 60 | 5,8 | 353,0 | 2 |
| 45 | Post-operative cognitive dysfunction at 3 months in adults after non-cardiac surgery: a qualitative systematic review | 60 | 5,8 | 397,0 | 6 |
| 46 | Prevalence and determinants of medication non-adherence in chronic pain patients: a systematic review | 60 | 5,8 | 373,0 | 3 |
| 47 | Real-time ultrasound-guided spinal anesthesia using the SonixGPS ultrasound guidance system: a feasibility study | 58 | 3,7 | 120,2 | 0 |
| 48 | Prophylactic tranexamic acid in parturients at low risk for post-partum haemorrhage: systematic review and meta-analysis | 58 | 3,5 | 237,3 | 6 |
| 49 | The analgesic effect of wound infiltration with local anaesthetics after breast surgery: a qualitative systematic review | 58 | 3,5 | 154,7 | 1 |
| 50 | Bystander first aid in trauma - prevalence and quality: a prospective observational study | 59 | 4,2 | 386,2 | 28 |
| 51 | Deep neuromuscular block reduces intra-abdominal pressure requirements during laparoscopic cholecystectomy: a prospective observational study | 59 | 4,2 | 191,8 | 2 |
| 52 | Efficacy and safety of pregabalin for treating painful diabetic peripheral neuropathy: a meta-analysis | 59 | 4,2 | 251,6 | 0 |
| 53 | Lower incidence of post-dural puncture headache with spinal catheterization after accidental dural puncture in obstetric patients | 58 | 3,3 | 202,5 | 2 |
| 54 | Routine pre-operative focused ultrasonography by anesthesiologists in patients undergoing urgent surgical procedures | 58 | 3,3 | 126,0 | 6 |
| 55 | Serum high-mobility group box 1 protein correlates with cognitive decline after gastrointestinal surgery | 58 | 3,3 | 90,3 | 1 |
| 56 | Positive end-expiratory pressure titration at bedside using electrical impedance tomography in post-operative cardiac surgery patients | 59 | 4,0 | 113,0 | 3 |
| 57 | Prevalence and predictors of persistent post-surgical pain 12months after thoracotomy | 59 | 4,0 | 146,2 | 8 |
| 58 | Veno-venous extracorporeal CO2 removal improves pulmonary hemodynamics in a porcine ARDS model | 59 | 4,0 | 82,8 | 0 |
| 59 | Acute kidney injury with hydroxyethyl starch 130/0.42 in severe sepsis | 59 | 4,0 | 134,4 | 4 |
| 60 | Is deep neuromuscular blockade beneficial in laparoscopic surgery? Yes, probably | 60 | 5,0 | 333,3 | 0 |
| 61 | Association of plasma chloride values with acute kidney injury in the critically ill - a prospective observational study | 60 | 5,0 | 147,3 | 0 |
| 62 | Nationwide incidence of serious complications of epidural analgesia in the United States | 60 | 5,0 | 329,0 | 4 |
| 63 | Intercostobrachial nerve handling and pain after axillary lymph node dissection for breast cancer | 58 | 3,2 | 72,3 | 1 |
| 64 | Recovery of gastrointestinal function with thoracic epidural vs. systemic analgesia following gastrointestinal surgery | 58 | 3,2 | 163,0 | 1 |
| 65 | Knee surgery recovery: Post-operative Quality of Recovery Scale comparison of age and complexity of surgery | 58 | 3,2 | 131,7 | 1 |
| 66 | The demise of early goal-directed therapy for severe sepsis and septic shock | 59 | 3,8 | 612,8 | 17 |
| 67 | Scandinavian clinical practice guideline on choice of fluid in resuscitation of critically ill patients with acute circulatory failure | 59 | 3,8 | 1224,4 | 27 |
| 68 | Scandinavian clinical practice guideline on mechanical ventilation in adults with the acute respiratory distress syndrome | 59 | 3,8 | 2617,2 | 6 |
| 69 | Microcirculatory dysfunction and tissue oxygenation in critical illness | 59 | 3,6 | 578,4 | 6 |
| 70 | In-hospital vs. 30-day mortality in the critically ill - a 2-year Swedish intensive care cohort analysis | 59 | 3,6 | 86,4 | 0 |
| 71 | Effect of unfractionated heparin on endothelial glycocalyx in a septic shock model | 59 | 3,6 | 165,8 | 1 |
| 72 | Predictive value of NGAL for use of renal replacement therapy in patients with severe sepsis | 59 | 3,6 | 145,8 | 3 |
| 73 | Helicopter-based emergency medical services for a sparsely populated region: A study of 42,500 dispatches | 60 | 4,5 | 204,5 | 2 |
| 74 | Intrathecal 1% 2-chloroprocaine vs. 0.5% bupivacaine in ambulatory surgery: a prospective, observer-blinded, randomised, controlled trial | 58 | 2,8 | 113,8 | 0 |
| 75 | Persistent opioid use and socio- economic factors: a population- based study in Norway | 58 | 2,8 | 112,2 | 0 |
| 76 | Assessment of acute stroke cerebral CT examinations by anaesthesiologists | 59 | 3,4 | 193,8 | 4 |
| 77 | Central venous pressure: we need to bring clinical use into physiological context | 59 | 3,4 | 433,6 | 2 |
| 78 | Post-anaesthetic emergence delirium in adults: incidence, predictors and consequences | 60 | 4,3 | 456,3 | 0 |
| 79 | Scandinavian clinical practice guideline on fluid and drug therapy in adults with acute respiratory distress syndrome | 60 | 4,3 | 1484,8 | 16 |
| 80 | A systematic review of the analgesic efficacy of cannabinoid medications in the management of acute pain | 61 | 5,7 | 1365,3 | 21 |
| 81 | Short general anaesthesia induces prolonged changes in gene expression in the mouse hippocampus | 58 | 2,7 | 65,3 | 0 |
| 82 | The effects of propofol vs. sevoflurane on post-operative pain and need of opioid | 58 | 2,7 | 97,3 | 3 |
| 83 | Pharmacokinetics of dexmedetomidine combined with therapeutic hypothermia in a piglet asphyxia model | 58 | 2,7 | 140,5 | 0 |
| 84 | Dabigatran anticoagulation and Stanford type A aortic dissection: lethal coincidence | 58 | 2,7 | 143,7 | 1 |
| 85 | Transversus abdominis plane (TAP) block after robot-assisted laparoscopic hysterectomy: a randomised clinical trial | 59 | 3,2 | 130,6 | 1 |
| 86 | Persistent postsurgical pain after video-assisted thoracic surgery - an observational study | 60 | 4,0 | 166,8 | 1 |
| 87 | Dehydration and fluid volume kinetics before major open abdominal surgery | 58 | 2,5 | 110,0 | 0 |
| 88 | Early screening to identify patients at risk of developing intra-abdominal hypertension and abdominal compartment syndrome | 58 | 2,5 | 98,7 | 3 |
| 89 | Concentrations of remifentanil, propofol, fentanyl, and midazolam during rewarming from therapeutic hypothermia | 58 | 2,5 | 102,3 | 0 |
| 90 | Post-operative atelectasis - a randomised trial investigating a ventilatory strategy and low oxygen fraction during recovery | 58 | 2,5 | 260,2 | 0 |
| 91 | Drowning in children: Utstein style reporting and outcome | 58 | 2,5 | 92,8 | 1 |
| 92 | Etomidate - a review of robust evidence for its use in various clinical scenarios | 58 | 2,5 | 280,5 | 4 |
| 93 | Drug-induced long QT syndrome and fatal arrhythmias in the intensive care unit | 58 | 2,5 | 318,5 | 0 |
| 94 | The association of perioperative dexamethasone, smoking and alcohol abuse with wound complications after laparotomy | 58 | 2,5 | 112,0 | 1 |
| 95 | The evidence of neuraxial administration of analgesics for cancer-related pain: a systematic review | 59 | 3,0 | 164,6 | 11 |
| 96 | Transversus abdominis plane block vs. wound infiltration in Caesarean section: a randomised controlled trial | 59 | 3,0 | 223,4 | 3 |
| 97 | Post-operative analgesia using intermittent vs. continuous adductor canal block technique: a randomized controlled trial | 60 | 3,8 | 206,5 | 0 |
| 98 | Aminocaproic acid administration is associated with reduced perioperative blood loss and transfusion in pediatric craniofacial surgery | 60 | 3,8 | 110,0 | 0 |
| 99 | Stress ulcer prophylaxis in the intensive care unit trial: detailed statistical analysis plan | 61 | 5,0 | 287,0 | 0 |
| 100 | Pain prevalence in hospitalized children: a prospective cross-sectional survey in four Danish university hospitals | 61 | 5,0 | 365,3 | 12 |

**Supplementary Table 2: Top 100 most downloaded papers in *Acta Anaesthesiologica Scandinavica* 2014-2018 sorted by total number of downlads.**

|  | Paper | Journal volume | Downloads per year | Citations per year | Altmetric Attention Score |
| --- | --- | --- | --- | --- | --- |
| 1 | Enhanced Recovery After Surgery (ERAS) for gastrointestinal surgery, part 1: pathophysiological considerations | 59 | 9582,8 | 17,0 | 3 |
| 2 | Enhanced Recovery After Surgery (ERAS) for gastrointestinal surgery, part 2: consensus statement for anaesthesia practice | 60 | 10506,3 | 36,0 | 7 |
| 3 | Scandinavian clinical practice guideline on mechanical ventilation in adults with the acute respiratory distress syndrome | 59 | 2617,2 | 3,8 | 6 |
| 4 | Fluid management in the critically ill: science or invention? | 60 | 2154,0 | 0,5 | 3 |
| 5 | Clinical guidelines on central venous catheterisation | 58 | 1165,2 | 10,3 | 10 |
| 6 | Scandinavian clinical practice guideline on choice of fluid in resuscitation of critically ill patients with acute circulatory failure | 59 | 1224,4 | 3,8 | 27 |
| 7 | Scandinavian SSAI clinical practice guideline on choice of inotropic agent for patients with acute circulatory failure | 62 | 3000,5 | 1,0 | 19 |
| 8 | Scandinavian clinical practice guideline on fluid and drug therapy in adults with acute respiratory distress syndrome | 60 | 1484,8 | 4,3 | 16 |
| 9 | Crew Resource Management in the Intensive Care Unit: a prospective 3‐year cohort study | 59 | 1089,4 | 4,6 | 52 |
| 10 | Scandinavian SSAI clinical practice guideline on pre‐hospital airway management | 60 | 1288,0 | 8,8 | 33 |
| 11 | Complex regional pain syndrome type I: a comprehensive review | 59 | 931,6 | 7,6 | 6 |
| 12 | Anaphylaxis during general anaesthesia: experience from a drug allergy centre in the UK | 61 | 1537,0 | 4,0 | 0 |
| 13 | Scandinavian SSAI clinical practice guideline on choice of first‐line vasopressor for patients with acute circulatory failure | 60 | 1152,3 | 3,5 | 14 |
| 14 | The effects of safety checklists in medicine: a systematic review | 58 | 718,8 | 8,5 | 11 |
| 15 | A systematic review of the analgesic efficacy of cannabinoid medications in the management of acute pain | 61 | 1365,3 | 5,7 | 21 |
| 16 | Post‐operative analgesic effects of paracetamol, NSAIDs, glucocorticoids, gabapentinoids and their combinations: a topical review | 58 | 652,0 | 10,3 | 1 |
| 17 | Emergency airway management – by whom and how? | 60 | 949,5 | 0,5 | 26 |
| 18 | New oral anticoagulants: clinical indications, monitoring and treatment of acute bleeding complications | 58 | 556,5 | 2,3 | 13 |
| 19 | Adverse effects of perioperative paracetamol, NSAIDs, glucocorticoids, gabapentinoids and their combinations: a topical review | 58 | 536,8 | 10,8 | 3 |
| 20 | The demise of early goal‐directed therapy for severe sepsis and septic shock | 59 | 612,8 | 3,8 | 17 |
| 21 | Anesthetic considerations in HELLP syndrome | 60 | 758,5 | 1,0 | 1 |
| 22 | A systematic review and meta‐analysis of ketamine for the prevention of persistent post‐surgical pain | 58 | 493,3 | 7,5 | 15 |
| 23 | Microcirculatory dysfunction and tissue oxygenation in critical illness | 59 | 578,4 | 3,6 | 6 |
| 24 | Gabapentin for post‐operative pain management – a systematic review with meta‐analyses and trial sequential analyses | 60 | 709,8 | 9,3 | 13 |
| 25 | Quality of post‐operative patient handover in the post‐anaesthesia care unit: a prospective analysis | 58 | 451,2 | 2,0 | 4 |
| 26 | Neuromuscular blockade for optimising surgical conditions during abdominal and gynaecological surgery: a systematic review | 59 | 514,8 | 10,0 | 0 |
| 27 | Inadvertent intrathecal injections and best practice management | 61 | 838,3 | 0,7 | 2 |
| 28 | Definition, prevalence, and outcome of feeding intolerance in intensive care: a systematic review and meta‐analysis | 58 | 403,3 | 8,3 | 2 |
| 29 | Total intravenous general anaesthesia vs. spinal anaesthesia for total hip arthroplasty: a randomised, controlled trial | 59 | 451,6 | 6,2 | 3 |
| 30 | Efficacy of simulation‐based trauma team training of non‐technical skills. A systematic review | 58 | 372,0 | 5,2 | 10 |
| 31 | Central venous pressure: we need to bring clinical use into physiological context | 59 | 433,6 | 3,4 | 2 |
| 32 | First‐aid training in school: amount, content and hindrances | 61 | 707,0 | 1,0 | 3 |
| 33 | Vancomycin levels are frequently subtherapeutic in critically ill patients: a prospective observational study | 61 | 675,7 | 3,3 | 4 |
| 34 | Sleep and delirium in unsedated patients in the intensive care unit | 60 | 484,8 | 2,3 | 22 |
| 35 | The role of prehabilitation in frail surgical patients: A systematic review | 62 | 966,5 | 1,5 | 2 |
| 36 | Bystander first aid in trauma – prevalence and quality: a prospective observational study | 59 | 386,2 | 4,2 | 28 |
| 37 | Drug‐induced long QT syndrome and fatal arrhythmias in the intensive care unit | 58 | 318,5 | 2,5 | 0 |
| 38 | Lung stress and strain calculations in mechanically ventilated patients in the intensive care unit | 60 | 474,5 | 2,0 | 1 |
| 39 | Dexmedetomidine for preventing sevoflurane‐related emergence agitation in children: a meta‐analysis of randomized controlled trials | 58 | 315,8 | 6,0 | 0 |
| 40 | The effects of surgical checklists on morbidity and mortality: a pre‐ and post‐intervention study | 59 | 367,8 | 2,6 | 1 |
| 41 | Post‐anaesthetic emergence delirium in adults: incidence, predictors and consequences | 60 | 456,3 | 4,3 | 0 |
| 42 | Intraoperative ketamine administration to prevent delirium or postoperative cognitive dysfunction: A systematic review and meta‐analysis | 62 | 891,0 | 1,5 | 5 |
| 43 | Ketamine and magnesium association reduces morphine consumption after scoliosis surgery: prospective randomised double‐blind study | 58 | 291,8 | 2,0 | 8 |
| 44 | Fluid responsiveness predicted by elevation of PEEP in patients with septic shock | 58 | 287,7 | 2,2 | 7 |
| 45 | Etomidate – a review of robust evidence for its use in various clinical scenarios | 58 | 280,5 | 2,5 | 4 |
| 46 | Long‐ or short‐acting opioids for chronic non‐malignant pain? A qualitative systematic review | 58 | 274,7 | 2,3 | 4 |
| 47 | Epidural analgesia in patients with traumatic rib fractures: a systematic review of randomised controlled trials | 59 | 327,0 | 2,0 | 13 |
| 48 | Intravenous ketamine during spinal and general anaesthesia for caesarean section: systematic review and meta‐analysis | 59 | 321,8 | 2,6 | 0 |
| 49 | Post‐operative cognitive dysfunction at 3 months in adults after non‐cardiac surgery: a qualitative systematic review | 60 | 397,0 | 5,8 | 6 |
| 50 | Fibrinogen concentrate for bleeding – a systematic review | 58 | 262,5 | 5,7 | 5 |
| 51 | Post‐operative atelectasis – a randomised trial investigating a ventilatory strategy and low oxygen fraction during recovery | 58 | 260,2 | 2,5 | 0 |
| 52 | Malignant hyperthermia, a Scandinavian update | 59 | 307,2 | 1,0 | 0 |
| 53 | Performance of Norwegian civilian EMTs and army medics in penetrating trauma: a controlled simulation‐based assessment | 61 | 512,0 | 0,0 | 31 |
| 54 | Major complications of epidural anesthesia: a prospective study of 5083 cases at a single hospital | 58 | 249,2 | 3,8 | 8 |
| 55 | Stress ulcer prophylaxis in the intensive care unit: an international survey of 97 units in 11 countries | 59 | 299,0 | 8,2 | 3 |
| 56 | Prevalence and determinants of medication non‐adherence in chronic pain patients: a systematic review | 60 | 373,0 | 5,8 | 3 |
| 57 | Assessing Nurse Anaesthetists' Non‐Technical Skills in the operating room | 58 | 244,0 | 5,0 | 1 |
| 58 | Ultrasound‐guided block of the suprascapular nerve – a volunteer study of a new proximal approach | 58 | 242,2 | 4,2 | 1 |
| 59 | The spread of injectate during saphenous nerve block at the adductor canal: a cadaver study | 59 | 288,2 | 7,0 | 1 |
| 60 | Is deep neuromuscular block beneficial in laparoscopic surgery? No, probably not | 60 | 357,8 | 6,0 | 0 |
| 61 | Prophylactic tranexamic acid in parturients at low risk for post‐partum haemorrhage: systematic review and meta‐analysis | 58 | 237,3 | 3,5 | 6 |
| 62 | Comparing efficacy and safety of fibrinogen concentrate to cryoprecipitate in bleeding patients: a systematic review | 60 | 353,0 | 5,8 | 2 |
| 63 | Norwegian survival prediction model in trauma: modelling effects of anatomic injury, acute physiology, age, and co‐morbidity | 58 | 231,8 | 4,2 | 3 |
| 64 | Nerve localization techniques for peripheral nerve block and possible future directions | 59 | 273,6 | 1,2 | 0 |
| 65 | Abstracts | 59 | 272,6 | 0,0 | 0 |
| 66 | Preserved oxygenation in obese patients receiving protective ventilation during laparoscopic surgery: a randomized controlled study | 60 | 338,0 | 3,3 | 3 |
| 67 | Effective dose of dexmedetomidine as an adjuvant sedative to peripheral nerve blockade in elderly patients | 62 | 674,0 | 1,0 | 0 |
| 68 | Is deep neuromuscular blockade beneficial in laparoscopic surgery? Yes, probably | 60 | 333,3 | 5,0 | 0 |
| 69 | Implementation of a trauma system in Norway: a national survey | 59 | 265,0 | 2,0 | 1 |
| 70 | Calculation of volatile anaesthetics consumption from agent concentration and fresh gas flow | 58 | 220,5 | 0,8 | 3 |
| 71 | Nationwide incidence of serious complications of epidural analgesia in the United States | 60 | 329,0 | 5,0 | 4 |
| 72 | Continuous positive airway pressure/pressure support pre‐oxygenation of morbidly obese patients | 58 | 218,3 | 4,0 | 1 |
| 73 | Post‐operative nausea and vomiting in bariatric surgery patients: an observational study | 61 | 435,0 | 3,0 | 3 |
| 74 | A comparison of sugammadex and neostigmine for reversal of rocuronium‐induced neuromuscular blockade in children | 61 | 434,3 | 1,3 | 2 |
| 75 | Propofol and survival: a meta‐analysis of randomized clinical trials | 59 | 260,6 | 1,8 | 1 |
| 76 | Tachyphylaxis to local anaesthetics. What is the clinical evidence? A systematic review | 60 | 325,8 | 0,8 | 0 |
| 77 | Assessment of skin temperature during regional anaesthesia—What the anaesthesiologist should know | 62 | 640,5 | 1,0 | 1 |
| 78 | Safety of the lateral trauma position in cervical spine injuries: a cadaver model study | 60 | 319,5 | 1,0 | 6 |
| 79 | Salbutamol has rapid onset pharmacodynamics as a bronchodilator | 60 | 319,0 | 0,3 | 1 |
| 80 | Efficacy and safety of pregabalin for treating painful diabetic peripheral neuropathy: a meta‐analysis | 59 | 251,6 | 4,2 | 0 |
| 81 | Paediatric ventilation treatment of acute lung injury in Nordic intensive care units | 59 | 245,0 | 0,4 | 2 |
| 82 | Lower incidence of post‐dural puncture headache with spinal catheterization after accidental dural puncture in obstetric patients | 58 | 202,5 | 3,3 | 2 |
| 83 | Chronic pre‐operative opioid use and acute pain after fast‐track total knee arthroplasty | 60 | 297,5 | 7,3 | 4 |
| 84 | Current evidence is not in support of lipid rescue therapy in local anaesthetic systemic toxicity | 60 | 297,3 | 0,8 | 2 |
| 85 | Effects of fluid restriction on measures of circulatory efficacy in adults with septic shock | 61 | 393,0 | 3,0 | 25 |
| 86 | Perioperative goal‐directed therapy: A systematic review without meta‐analysis | 62 | 589,0 | 4,0 | 2 |
| 87 | Parecoxib, propacetamol, and their combination for analgesia after total hip arthroplasty: a randomized non‐inferiority trial | 61 | 391,3 | 1,7 | 0 |
| 88 | Ultrasonography‐guided radial artery catheterization is superior compared with the traditional palpation technique | 58 | 195,2 | 6,2 | 0 |
| 89 | Medical emergency team activation: performance of conventional dichotomised criteria versus national early warning score | 58 | 193,8 | 4,0 | 0 |
| 90 | Mortality in elderly ICU patients: a cohort study | 58 | 191,0 | 6,7 | 0 |
| 91 | Post‐discharge nausea and vomiting after total intravenous anaesthesia and standardised PONV prophylaxis for ambulatory surgery | 61 | 379,3 | 1,0 | 0 |
| 92 | Infrared Red Intubation System (IRRIS) guided flexile videoscope assisted difficult airway management | 62 | 565,5 | 2,5 | 12 |
| 93 | Analgesia Nociception Index (ANI) to predict intraoperative haemodynamic changes: results of a pilot investigation | 58 | 186,5 | 4,0 | 1 |
| 94 | Transversus abdominis plane block vs. wound infiltration in Caesarean section: a randomised controlled trial | 59 | 223,4 | 3,0 | 3 |
| 95 | Pain prevalence in hospitalized children: a prospective cross‐sectional survey in four Danish university hospitals | 61 | 365,3 | 5,0 | 12 |
| 96 | Reducing pre‐operative fasting while preserving operating room scheduling flexibility: feasibility and impact on patient discomfort | 60 | 272,3 | 0,5 | 0 |
| 97 | Optimising abdominal space with deep neuromuscular blockade in gynaecologic laparoscopy – a randomised, blinded crossover study | 59 | 217,0 | 5,8 | 1 |
| 98 | The ultrasound‐assisted paraspinous approach to lumbar neuraxial blockade: a simplified technique in patients with difficult anatomy | 59 | 209,6 | 1,0 | 0 |
| 99 | Norepinephrine reduces arterial compliance less than phenylephrine when treating general anesthesia‐induced arterial hypotension | 61 | 348,0 | 1,3 | 0 |
| 100 | Indocyanine green plasma disappearance rate as dynamic liver function test in critically ill patients | 58 | 170,5 | 5,0 | 1 |

**Supplementary Table 3: Top 100 most cited papers in *Anesthesia & Analgesia* 2014-2018 sorted by total number of citations.**

|  | Paper | Publication year | Citations per year | Downloads per year | Altmetric Attention Score |
| --- | --- | --- | --- | --- | --- |
| 1 | Consensus Guidelines for the Management of Postoperative Nausea and Vomiting | 2014 | 83,83 | 11400,50 | 32 |
| 2 | Reduced Length of Hospital Stay in Colorectal Surgery after Implementation of an Enhanced Recovery Protocol | 2014 | 27,33 | 1235,67 | 46 |
| 3 | Outcomes for Extremely Premature Infants | 2015 | 24,20 | 686,20 | 134 |
| 4 | The Perioperative Surgical Home as a Future Perioperative Practice Model | 2014 | 18,17 | 1531,67 | 2 |
| 5 | Transversus Abdominis Plane Block to Ameliorate Postoperative Pain Outcomes After Laparoscopic Surgery: A Meta-Analysis of Randomized Controlled Trials | 2014 | 16,17 | 592,33 | 1 |
| 6 | The Impact of Sleep Apnea on Postoperative Utilization of Resources and Adverse Outcomes | 2014 | 15,83 | 261,17 | 4 |
| 7 | The Perioperative Surgical Home: How Anesthesiology Can Collaboratively Achieve and Leverage the Triple Aim in Health Care | 2014 | 15,67 | 551,33 | 0 |
| 8 | Society of Anesthesia and Sleep Medicine Guidelines on Preoperative Screening and Assessment of Adult Patients With Obstructive Sleep Apnea | 2016 | 22,50 | 2612,50 | 19 |
| 9 | Lung Injury After One-Lung Ventilation: A Review of the Pathophysiologic Mechanisms Affecting the Ventilated and the Collapsed Lung | 2015 | 16,40 | 1070,00 | 2 |
| 10 | Guidelines for Performing a Comprehensive Transesophageal Echocardiographic Examination: Recommendations from the American Society of Echocardiography and the Society of Cardiovascular Anesthesiologists | 2014 | 13,50 | 978,00 | 10 |
| 11 | The Analgesic Efficacy of Ultrasound-Guided Transversus Abdominis Plane Block in Adult Patients: A Meta-Analysis | 2015 | 16,00 | 439,33 | 7 |
| 12 | Surgical Space Conditions During Low-Pressure Laparoscopic Cholecystectomy with Deep Versus Moderate Neuromuscular Blockade: A Randomized Clinical Study | 2014 | 13,00 | 367,00 | 0 |
| 13 | Postoperative Hypoxemia Is Common and Persistent: A Prospective Blinded Observational Study | 2015 | 15,40 | 385,80 | 12 |
| 14 | Death or Neurologic Injury after Tonsillectomy in Children with a Focus on Obstructive Sleep Apnea: Houston, We Have a Problem! | 2014 | 12,83 | 556,67 | 8 |
| 15 | Implementation of a Total Joint Replacement- Focused Perioperative Surgical Home: A Management Case Report | 2014 | 12,67 | 607,83 | 10 |
| 16 | Extracorporeal Membrane Oxygenation in the Adult: A Review of Anticoagulation Monitoring and Transfusion | 2014 | 12,67 | 896,83 | 0 |
| 17 | Postoperative Delirium in a Substudy of Cardiothoracic Surgical Patients in the BAG- RECALL Clinical Trial | 2014 | 12,67 | 249,17 | 4 |
| 18 | Does Obstructive Sleep Apnea Influence Perioperative Outcome? A Qualitative Systematic Review for the Society of Anesthesia and Sleep Medicine Task Force on Preoperative Preparation of Patients with Sleep-Disordered Breathing | 2016 | 18,75 | 554,75 | 2 |
| 19 | Correlation Coefficients: Appropriate Use and Interpretation | 2018 | 36,50 | 13035,50 | 12 |
| 20 | Perioperative Goal-Directed Hemodynamic Optimization Using Noninvasive Cardiac Output Monitoring in Major Abdominal Surgery: A Prospective, Randomized, Multicenter, Pragmatic Trial: POEMAS Study (PeriOperative goal-directed thErapy in Major Abdominal Surgery) | 2014 | 12,17 | 553,50 | 4 |
| 21 | A General Purpose Pharmacokinetic Model for Propofol | 2014 | 11,83 | 361,67 | 2 |
| 22 | Intraoperative Electroencephalogram Suppression Predicts Postoperative Delirium | 2016 | 17,50 | 382,00 | 10 |
| 23 | The Long-Term Impact of Early Cardiovascular Therapy Intensification for Postoperative Troponin Elevation After Major Vascular Surgery | 2014 | 11,67 | 248,33 | 3 |
| 24 | The Effects of Perineural Versus Intravenous Dexamethasone on Sciatic Nerve Blockade Outcomes: A Randomized, Double-Blind, Placebo-Controlled Study | 2014 | 11,17 | 344,17 | 3 |
| 25 | Continuous Ultrasound-Guided Adductor Canal Block for Total Knee Arthroplasty: A Randomized, Double-Blind Trial | 2014 | 11,00 | 530,17 | 11 |
| 26 | Continuous Peripheral Nerve Blocks: An Update of the Published Evidence and Comparison With Novel, Alternative Analgesic Modalities | 2017 | 21,00 | 1125,00 | 2 |
| 27 | Will the Real Benefits of Single-Shot Interscalene Block Please Stand Up? A Systematic Review and Meta-Analysis | 2015 | 12,60 | 504,20 | 15 |
| 28 | Strategies for Net Cost Reductions with the Expanded Role and Expertise of Anesthesiologists in the Perioperative Surgical Home | 2014 | 10,50 | 271,50 | 0 |
| 29 | RETRACTED: The Impact of Anesthesiologists on Coronary Artery Bypass Graft Surgery Outcomes (Retracted article. See vol. 122, pg. 1730, 2016) | 2015 | 12,40 | 1593,60 | 37 |
| 30 | The RECITE Study: A Canadian Prospective, Multicenter Study of the Incidence and Severity of Residual Neuromuscular Blockade | 2015 | 12,00 | 617,00 | 6 |
| 31 | Chronic Opioid Use and Central Sleep Apnea: A Review of the Prevalence, Mechanisms, and Perioperative Considerations | 2015 | 12,00 | 565,80 | 6 |
| 32 | The Society for Obstetric Anesthesia and Perinatology Consensus Statement on the Management of Cardiac Arrest in Pregnancy | 2014 | 10,00 | 814,33 | 9 |
| 33 | Local Anesthetics Induce Apoptosis in Human Breast Tumor Cells | 2014 | 9,33 | 243,00 | 0 |
| 34 | The Effects of Continuous Positive Airway Pressure on Postoperative Outcomes in Obstructive Sleep Apnea Patients Undergoing Surgery: A Systematic Review and Meta-Analysis | 2015 | 10,60 | 467,00 | 1 |
| 35 | Are Caudal Blocks for Pain Control Safe in Children? An Analysis of 18,650 Caudal Blocks from the Pediatric Regional Anesthesia Network (PRAN) Database | 2015 | 10,60 | 532,80 | 18 |
| 36 | A Randomized Controlled Trial of the Efficacy and Respiratory Effects of Patient-Controlled Intravenous Remifentanil Analgesia and Patient-Controlled Epidural Analgesia in Laboring Women | 2014 | 8,83 | 407,17 | 4 |
| 37 | Trends in Tramadol: Pharmacology, Metabolism, and Misuse | 2017 | 17,33 | 1935,67 | 8 |
| 38 | Malignant Hyperthermia in Canada: Characteristics of Index Anesthetics in 129 Malignant Hyperthermia Susceptible Probands | 2014 | 8,67 | 296,50 | 16 |
| 39 | Tracking Changes in Cardiac Output: Statistical Considerations on the 4-Quadrant Plot and the Polar Plot Methodology | 2015 | 10,20 | 163,80 | 0 |
| 40 | Portable Infrared Pupillometry: A Review | 2015 | 10,20 | 509,80 | 1 |
| 41 | Crystalloids Versus Colloids: Exploring Differences in Fluid Requirements by Systematic Review and Meta-Regression | 2015 | 10,20 | 735,20 | 3 |
| 42 | The WFSA Global Anesthesia Workforce Survey | 2017 | 16,67 | 712,33 | 68 |
| 43 | Cognitive Dysfunction After Fast-Track Hip and Knee Replacement | 2014 | 8,33 | 353,67 | 2 |
| 44 | The Pathway of Injectate Spread With the Transmuscular Quadratus Lumborum Block: A Cadaver Study | 2017 | 16,33 | 512,00 | 8 |
| 45 | Laparoscopic Surgery and Muscle Relaxants: Is Deep Block Helpful? | 2015 | 9,80 | 921,60 | 1 |
| 46 | A Review of Opioid-Sparing Modalities in Perioperative Pain Management: Methods to Decrease Opioid Use Postoperatively | 2017 | 16,00 | 1921,33 | 5 |
| 47 | Preoxygenation: Physiologic Basis, Benefits, and Potential Risks | 2017 | 16,00 | 3998,67 | 10 |
| 48 | Pain After Unilateral Total Knee Arthroplasty: A Prospective Randomized Controlled Trial Examining the Analgesic Effectiveness of a Combined Adductor Canal Peripheral Nerve Block with Periarticular Infiltration Versus Adductor Canal Nerve Block Alone Versus Periarticular Infiltration Alone | 2016 | 12,00 | 509,75 | 12 |
| 49 | Ultrasound-Guided Thoracic Paravertebral Blockade: A Retrospective Study of the Incidence of Complications | 2016 | 12,00 | 414,00 | 15 |
| 50 | Regional Versus General Anesthesia in Surgical Patients with Chronic Obstructive Pulmonary Disease: Does Avoiding General Anesthesia Reduce the Risk of Postoperative Complications? | 2015 | 9,60 | 695,60 | 4 |
| 51 | Setting Individualized Positive End- Expiratory Pressure Level with a Positive End- Expiratory Pressure Decrement Trial After a Recruitment Maneuver Improves Oxygenation and Lung Mechanics During One- Lung Ventilation | 2014 | 8,00 | 383,50 | 7 |
| 52 | Nitrous Oxide for the Management of Labor Pain: A Systematic Review | 2014 | 8,00 | 1518,50 | 25 |
| 53 | Impact of Enhanced Recovery After Surgery and Opioid-Free Anesthesia on Opioid Prescriptions at Discharge From the Hospital: A Historical-Prospective Study | 2017 | 15,67 | 1047,67 | 16 |
| 54 | Assessing the Methodology for Calculating Platelet Contribution to Clot Strength (Platelet Component) in Thromboelastometry and Thrombelastography | 2015 | 9,40 | 198,80 | 0 |
| 55 | Chronic Opioid Use After Surgery: Implications for Perioperative Management in the Face of the Opioid Epidemic | 2017 | 15,33 | 1157,00 | 20 |
| 56 | An Evaluation of a Zero-Heat-Flux Cutaneous Thermometer in Cardiac Surgical Patients | 2014 | 7,50 | 139,67 | 4 |
| 57 | Systematic Reviews of Anesthesiologic Interventions Reported as Statistically Significant: Problems with Power, Precision, and Type 1 Error Protection | 2015 | 8,80 | 92,80 | 2 |
| 58 | An Exploratory Cohort Study Comparing Prothrombin Complex Concentrate and Fresh Frozen Plasma for the Treatment of Coagulopathy After Complex Cardiac Surgery | 2015 | 8,80 | 303,40 | 2 |
| 59 | The Incidence and Risk Factors for Perioperative Cardiac Arrest Observed in the National Anesthesia Clinical Outcomes Registry | 2015 | 8,80 | 616,40 | 5 |
| 60 | Malignant Hyperthermia Deaths Related to Inadequate Temperature Monitoring, 2007-2012: A Report from The North American Malignant Hyperthermia Registry of the Malignant Hyperthermia Association of the United States | 2014 | 7,33 | 366,67 | 16 |
| 61 | Intraoperative Dexamethasone and Delirium After Cardiac Surgery: A Randomized Clinical Trial | 2014 | 7,33 | 272,50 | 2 |
| 62 | Sedation Depth During Spinal Anesthesia and Survival in Elderly Patients Undergoing Hip Fracture Repair | 2014 | 7,17 | 327,67 | 9 |
| 63 | Accuracy of Continuous Noninvasive Hemoglobin Monitoring: A Systematic Review and Meta-Analysis | 2014 | 7,00 | 245,00 | 0 |
| 64 | A Prospective Comparison of a Noninvasive Cardiac Output Monitor Versus Esophageal Doppler Monitor for Goal-Directed Fluid Therapy in Colorectal Surgery Patients | 2014 | 7,00 | 337,50 | 8 |
| 65 | The Efficacy of Antifibrinolytic Drugs in Children Undergoing Noncardiac Surgery: A Systematic Review of the Literature | 2014 | 7,00 | 321,00 | 4 |
| 66 | Increased Glycemic Variability in Patients with Elevated Preoperative HbA1C Predicts Adverse Outcomes Following Coronary Artery Bypass Grafting Surgery | 2014 | 7,00 | 197,00 | 5 |
| 67 | Neuraxial Anesthesia for the Prevention of Postoperative Mortality and Major Morbidity: An Overview of Cochrane Systematic Reviews | 2014 | 6,83 | 455,83 | 8 |
| 68 | Transversus Abdominis Plane Block in Children: A Multicenter Safety Analysis of 1994 Cases from the PRAN (Pediatric Regional Anesthesia Network) Database | 2014 | 6,83 | 249,50 | 3 |
| 69 | The Effectiveness of Different Functional Fibrinogen Polymerization Assays in Eliminating Platelet Contribution to Clot Strength in Thromboelastometry | 2014 | 6,83 | 122,83 | 0 |
| 70 | Risk Factors for Opioid-Use Disorder and Overdose | 2017 | 13,33 | 1796,33 | 25 |
| 71 | Perioperative Ultrasound Training in Anesthesiology: A Call to Action | 2016 | 10,00 | 462,25 | 1 |
| 72 | Obstetric Anesthesia Workforce Survey: A 30-Year Update | 2016 | 10,00 | 261,50 | 0 |
| 73 | Rationale and Design of the Balanced Anesthesia Study: A Prospective Randomized Clinical Trial of Two Levels of Anesthetic Depth on Patient Outcome After Major Surgery | 2015 | 8,00 | 343,60 | 4 |
| 74 | First-Attempt Intubation Success of Video Laryngoscopy in Patients with Anticipated Difficult Direct Laryngoscopy: A Multicenter Randomized Controlled Trial Comparing the C-MAC D-Blade Versus the GlideScope in a Mixed Provider and Diverse Patient Population | 2016 | 9,75 | 490,50 | 3 |
| 75 | Difficult Airway Response Team: A Novel Quality Improvement Program for Managing Hospital- Wide Airway Emergencies | 2015 | 7,80 | 352,00 | 18 |
| 76 | Epidural Labor Analgesia Is Associated with a Decreased Risk of Postpartum Depression: A Prospective Cohort Study | 2014 | 6,50 | 684,50 | 64 |
| 77 | The Frequency of Cardiac Arrests in Patients with Congenital Heart Disease Undergoing Cardiac Catheterization | 2014 | 6,50 | 202,83 | 1 |
| 78 | The Risk of Falls After Total Knee Arthroplasty with the Use of a Femoral Nerve Block Versus an Adductor Canal Block: A Double-Blinded Randomized Controlled Study | 2016 | 9,50 | 436,75 | 12 |
| 79 | Dexmedetomidine Reduces Propofol and Remifentanil Requirements During Bispectral Index- Guided Closed- Loop Anesthesia: A Double- Blind, Placebo-Controlled Trial | 2014 | 6,33 | 295,00 | 9 |
| 80 | A Decrease in Spatially Resolved Near-Infrared Spectroscopy-Determined Frontal Lobe Tissue Oxygenation by Phenylephrine Reflects Reduced Skin Blood Flow | 2014 | 6,33 | 113,33 | 0 |
| 81 | Consensus Statement on Perioperative Use of Neuromuscular Monitoring | 2018 | 18,50 | 1671,00 | 10 |
| 82 | The US Opioid Crisis: Current Federal and State Legal Issues | 2017 | 12,33 | 1990,00 | 5 |
| 83 | Target-Controlled Infusion: A Mature Technology | 2016 | 9,25 | 642,00 | 6 |
| 84 | Perioperative Complications in Obstructive Sleep Apnea Patients Undergoing Surgery: A Review of the Legal Literature | 2016 | 9,25 | 747,25 | 0 |
| 85 | National Partnership for Maternal Safety: Consensus Bundle on Obstetric Hemorrhage | 2015 | 7,40 | 838,60 | 18 |
| 86 | Extracorporeal Membrane Oxygenation Induces Short-Term Loss of High-Molecular-Weight von Willebrand Factor Multimers | 2015 | 7,40 | 168,20 | 0 |
| 87 | The Implementation of Quantitative Electromyographic Neuromuscular Monitoring in an Academic Anesthesia Department | 2014 | 6,17 | 263,33 | 0 |
| 88 | A Randomized Controlled Trial of Variable Rate Phenylephrine Infusion With Rescue Phenylephrine Boluses Versus Rescue Boluses Alone on Physician Interventions During Spinal Anesthesia for Elective Cesarean Delivery | 2014 | 6,17 | 312,00 | 1 |
| 89 | The Relationship of Body Mass Index with the Incidence of Postdural Puncture Headache in Parturients | 2015 | 7,20 | 391,80 | 2 |
| 90 | Shoulder Surgery in the Beach Chair Position Is Associated with Diminished Cerebral Autoregulation but No Differences in Postoperative Cognition or Brain Injury Biomarker Levels Compared with Supine Positioning: The Anesthesia Patient Safety Foundation Beach Chair Study | 2015 | 7,20 | 450,20 | 5 |
| 91 | The Use of Pulse Pressure Variation and Stroke Volume Variation in Spontaneously Breathing Patients to Assess Dynamic Arterial Elastance and to Predict Arterial Pressure Response to Fluid Administration | 2015 | 7,20 | 609,80 | 3 |
| 92 | An Acetate-Buffered Balanced Crystalloid Versus 0.9% Saline in Patients with End-Stage Renal Disease Undergoing Cadaveric Renal Transplantation: A Prospective Randomized Controlled Trial | 2015 | 7,20 | 358,40 | 13 |
| 93 | Prediction of Intraoperative Transfusion Requirements During Orthotopic Liver Transplantation and the Influence on Postoperative Patient Survival | 2014 | 6,00 | 160,17 | 0 |
| 94 | Disconnecting Consciousness: Is There a Common Anesthetic End Point? | 2016 | 8,50 | 280,25 | 99 |
| 95 | A Systematic Review and Meta-Analysis of the Global Burden of Chronic Pain Without Clear Etiology in Low and Middle-Income Countries: Trends in Heterogeneous Data and a Proposal for New Assessment Methods | 2016 | 8,50 | 339,00 | 12 |
| 96 | Postoperative Morbidity and Discharge Destinations After Fast-Track Hip and Knee Arthroplasty in Patients Older Than 85 Years | 2016 | 8,50 | 302,00 | 0 |
| 97 | Intraoperative Lung-Protective Ventilation Trends and Practice Patterns: A Report from the Multicenter Perioperative Outcomes Group | 2015 | 6,80 | 289,40 | 8 |
| 98 | Performance of Propofol Target-Controlled Infusion Models in the Obese: Pharmacokinetic and Pharmacodynamic Analysis | 2014 | 5,67 | 366,33 | 2 |
| 99 | National Pediatric Anesthesia Safety Quality Improvement Program in the United States | 2014 | 5,67 | 271,67 | 2 |
| 100 | The Effectiveness of Cricoid Pressure for Occluding the Esophageal Entrance in Anesthetized and Paralyzed Patients: An Experimental and Observational Glidescope Study | 2014 | 5,67 | 493,33 | 27 |

**Supplementary Table 4: Top 100 most downloaded papers in *Anesthesia & Analgesia* 2014-2018 sorted by total number of downloads.**

|  | Paper | Publication year | Downloads per year | Citations per year | Altmetric Attention Score |
| --- | --- | --- | --- | --- | --- |
| 1 | Consensus Guidelines for the Management of Postoperative Nausea and Vomiting. | 2014 | 11400,50 | 83,83 | 32 |
| 2 | Correlation Coefficients: Appropriate Use and Interpretation. | 2018 | 13035,50 | 36,50 | 12 |
| 3 | Cardiac Arrest in the Operating Room: Resuscitation and Management for the Anesthesiologist: Part 1. | 2018 | 8452,50 | 6,50 | 45 |
| 4 | Multimodal General Anesthesia: Theory and Practice. | 2018 | 6763,50 | 8,00 | 46 |
| 5 | Preoxygenation: Physiologic Basis, Benefits, and Potential Risks. | 2017 | 3998,67 | 16,00 | 10 |
| 6 | Society of Anesthesia and Sleep Medicine Guidelines on Preoperative Screening and Assessment of Adult Patients With Obstructive Sleep Apnea. | 2016 | 2612,50 | 22,50 | 19 |
| 7 | Methylene Blue: Magic Bullet for Vasoplegia?. | 2016 | 2591,75 | 7,50 | 13 |
| 8 | Hypertrophic Cardiomyopathy: A Review. | 2015 | 1901,00 | 5,80 | 2 |
| 9 | The Perioperative Surgical Home as a Future Perioperative Practice Model. | 2014 | 1531,67 | 18,17 | 2 |
| 10 | Nitrous Oxide for the Management of Labor Pain: A Systematic Review. | 2014 | 1518,50 | 8,00 | 25 |
| 11 | Cardiac Arrest in the Operating Room: Part 2-Special Situations in the Perioperative Period. | 2018 | 4341,50 | 3,50 | 28 |
| 12 | Anesthetic Management During Cardiopulmonary Bypass: A Systematic Review. | 2015 | 1649,80 | 3,60 | 2 |
| 13 | The Impact of Anesthesiologists on Coronary Artery Bypass Graft Surgery Outcomes. | 2015 | 1593,60 | 12,40 | 37 |
| 14 | Survival Analysis and Interpretation of Time-to-Event Data: The Tortoise and the Hare. | 2018 | 3940,50 | 1,00 | 1 |
| 15 | Estimated Maximal Safe Dosages of Tumescent Lidocaine. | 2016 | 1855,50 | 6,25 | 6 |
| 16 | Reduced Length of Hospital Stay in Colorectal Surgery after Implementation of an Enhanced Recovery Protocol. | 2014 | 1235,67 | 27,33 | 46 |
| 17 | The Society for Obstetric Anesthesia and Perinatology Consensus Statement on the Anesthetic Management of Pregnant and Postpartum Women Receiving Thromboprophylaxis or Higher Dose Anticoagulants. | 2018 | 3628,00 | 13,50 | 74 |
| 18 | Fontan Physiology Revisited. | 2015 | 1214,60 | 4,00 | 4 |
| 19 | The US Opioid Crisis: Current Federal and State Legal Issues. | 2017 | 1990,00 | 12,33 | 5 |
| 20 | Guidelines for Performing a Comprehensive Transesophageal Echocardiographic Examination: Recommendations from the American Society of Echocardiography and the Society of Cardiovascular Anesthesiologists. | 2014 | 978,00 | 13,50 | 10 |
| 21 | Trends in Tramadol: Pharmacology, Metabolism, and Misuse. | 2017 | 1935,67 | 17,33 | 8 |
| 22 | A Review of Opioid-Sparing Modalities in Perioperative Pain Management: Methods to Decrease Opioid Use Postoperatively. | 2017 | 1921,33 | 16,00 | 5 |
| 23 | Perioperative Acute Kidney Injury: An Under-Recognized Problem. | 2017 | 1854,67 | 6,00 | 2 |
| 24 | Continuous Pulse Oximetry and Capnography Monitoring for Postoperative Respiratory Depression and Adverse Events: A Systematic Review and Meta-analysis. | 2017 | 1833,67 | 5,67 | 5 |
| 25 | Society of Anesthesia and Sleep Medicine Guideline on Intraoperative Management of Adult Patients With Obstructive Sleep Apnea. | 2018 | 2714,00 | 8,50 | 19 |
| 26 | Risk Factors for Opioid-Use Disorder and Overdose. | 2017 | 1796,33 | 13,33 | 25 |
| 27 | Extracorporeal Membrane Oxygenation in the Adult: A Review of Anticoagulation Monitoring and Transfusion. | 2014 | 896,83 | 12,67 | 0 |
| 28 | Lung Injury After One-Lung Ventilation: A Review of the Pathophysiologic Mechanisms Affecting the Ventilated and the Collapsed Lung. | 2015 | 1070,00 | 16,40 | 2 |
| 29 | Update on Perioperative Acute Kidney Injury. | 2018 | 2663,50 | 3,00 | 9 |
| 30 | Preoperative Fasting Guidelines: Why Are We Not Following Them?: The Time to Act Is NOW. | 2017 | 1775,33 | 1,67 | 5 |
| 31 | The History of Target-Controlled Infusion. | 2016 | 1304,25 | 8,25 | 6 |
| 32 | Dural Puncture Epidural Technique Improves Labor Analgesia Quality With Fewer Side Effects Compared With Epidural and Combined Spinal Epidural Techniques: A Randomized Clinical Trial. | 2017 | 1719,00 | 9,00 | 15 |
| 33 | The Physiology of Cardiopulmonary Resuscitation. | 2016 | 1287,00 | 7,00 | 14 |
| 34 | Gastric Tubes and Airway Management in Patients at Risk of Aspiration: History, Current Concepts, and Proposal of an Algorithm. | 2014 | 832,00 | 2,67 | 2 |
| 35 | The Society for Obstetric Anesthesia and Perinatology Consensus Statement on the Management of Cardiac Arrest in Pregnancy. | 2014 | 814,33 | 10,00 | 9 |
| 36 | Pierre Robin Sequence: A Perioperative Review. | 2014 | 805,33 | 2,00 | 8 |
| 37 | A Systematic Review of Outcomes Associated With Withholding or Continuing Angiotensin-Converting Enzyme Inhibitors and Angiotensin Receptor Blockers Before Noncardiac Surgery. | 2018 | 2359,50 | 9,00 | 42 |
| 38 | Perioperative Peripheral Nerve Injury After General Anesthesia: A Qualitative Systematic Review. | 2018 | 2358,00 | 1,50 | 23 |
| 39 | Postoperative Cognitive Dysfunction and Noncardiac Surgery. | 2018 | 2313,00 | 6,50 | 7 |
| 40 | Laparoscopic Surgery and Muscle Relaxants: Is Deep Block Helpful?. | 2015 | 921,60 | 9,80 | 1 |
| 41 | Take It or Leave It: A Meta-analysis of Perioperative ACE Inhibitors and ARBs. | 2018 | 2297,50 | 0,00 | 3 |
| 42 | American Society for Enhanced Recovery and Perioperative Quality Initiative Joint Consensus Statement on Nutrition Screening and Therapy Within a Surgical Enhanced Recovery Pathway. | 2018 | 2247,00 | 16,00 | 393 |
| 43 | Blood Pressure Monitoring for the Anesthesiologist: A Practical Review. | 2016 | 1114,75 | 5,50 | 1 |
| 44 | MicroRNAs as Clinical Biomarkers and Therapeutic Tools in Perioperative Medicine. | 2018 | 2153,00 | 8,00 | 1 |
| 45 | Optimal Ventilation of the Anesthetized Pediatric Patient. | 2015 | 856,60 | 3,00 | 6 |
| 46 | Statistical Significance Versus Clinical Importance of Observed Effect Sizes: What Do P Values and Confidence Intervals Really Represent?. | 2018 | 2115,50 | 12,50 | 16 |
| 47 | National Partnership for Maternal Safety: Consensus Bundle on Obstetric Hemorrhage. | 2015 | 838,60 | 7,40 | 18 |
| 48 | Epidural Labor Analgesia Is Associated with a Decreased Risk of Postpartum Depression: A Prospective Cohort Study. | 2014 | 684,50 | 6,50 | 64 |
| 49 | Hyperbaric Versus Plain Bupivacaine for Spinal Anesthesia for Cesarean Delivery. | 2015 | 805,80 | 0,20 | 1 |
| 50 | Should Norepinephrine, Rather than Phenylephrine, Be Considered the Primary Vasopressor in Anesthetic Practice?. | 2016 | 1003,75 | 3,50 | 132 |
| 51 | Clinical Research Methodology 1: Study Designs and Methodologic Sources of Error. | 2015 | 765,60 | 1,80 | 4 |
| 52 | Stewart Acid-Base: A Simplified Bedside Approach. | 2016 | 939,75 | 2,50 | 2 |
| 53 | Dr. Virginia Apgar and the Apgar Score: How the Apgar Score Came to Be. | 2015 | 744,60 | 0,20 | 13 |
| 54 | The Analgesic Effect of Ultrasound-Guided Quadratus Lumborum Block After Cesarean Delivery: A Randomized Clinical Trial. | 2018 | 1844,00 | 9,50 | 44 |
| 55 | Crystalloids Versus Colloids: Exploring Differences in Fluid Requirements by Systematic Review and Meta-Regression. | 2015 | 735,20 | 10,20 | 3 |
| 56 | The Effect of Single Low-Dose Dexamethasone on Blood Glucose Concentrations in the Perioperative Period: A Randomized, Placebo-Controlled Investigation in Gynecologic Surgical Patients. | 2014 | 611,00 | 3,50 | 1 |
| 57 | Misconceptions Surrounding Penicillin Allergy: Implications for Anesthesiologists. | 2018 | 1825,00 | 2,00 | 165 |
| 58 | Implementation of a Total Joint Replacement-Focused Perioperative Surgical Home: A Management Case Report. | 2014 | 607,83 | 12,67 | 10 |
| 59 | Trauma Bleeding Management: The Concept of Goal-Directed Primary Care. | 2014 | 607,33 | 5,50 | 5 |
| 60 | Enhanced Recovery after Surgery Versus Perioperative Surgical Home: Is It All in the Name?. | 2014 | 603,83 | 4,17 | 0 |
| 61 | Medical Advances in the Treatment of Postpartum Hemorrhage. | 2014 | 596,50 | 3,50 | 8 |
| 62 | Transversus Abdominis Plane Block to Ameliorate Postoperative Pain Outcomes After Laparoscopic Surgery: A Meta-Analysis of Randomized Controlled Trials. | 2014 | 592,33 | 16,17 | 1 |
| 63 | Addition of Neostigmine and Atropine to Conventional Management of Postdural Puncture Headache: A Randomized Controlled Trial. | 2018 | 1755,50 | 2,50 | 14 |
| 64 | Regional Versus General Anesthesia in Surgical Patients with Chronic Obstructive Pulmonary Disease: Does Avoiding General Anesthesia Reduce the Risk of Postoperative Complications?. | 2015 | 695,60 | 9,60 | 4 |
| 65 | Chronic Opioid Use After Surgery: Implications for Perioperative Management in the Face of the Opioid Epidemic. | 2017 | 1157,00 | 15,33 | 20 |
| 66 | Neuraxial Anesthesia in Parturients with Thrombocytopenia: A Multisite Retrospective Cohort Study. | 2015 | 693,00 | 4,00 | 1 |
| 67 | Outcomes for Extremely Premature Infants. | 2015 | 686,20 | 24,20 | 134 |
| 68 | Using Integrative Medicine in Pain Management: An Evaluation of Current Evidence. | 2017 | 1137,00 | 2,67 | 117 |
| 69 | Ventricular Tachycardia Ablation: A Comprehensive Review for Anesthesiologists. | 2015 | 682,20 | 0,20 | 1 |
| 70 | Continuous Peripheral Nerve Blocks: An Update of the Published Evidence and Comparison With Novel, Alternative Analgesic Modalities. | 2017 | 1125,00 | 21,00 | 2 |
| 71 | Death or Neurologic Injury after Tonsillectomy in Children with a Focus on Obstructive Sleep Apnea: Houston, We Have a Problem! | 2014 | 556,67 | 12,83 | 8 |
| 72 | Review of the Alternatives to Epidural Blood Patch for Treatment of Postdural Puncture Headache in the Parturient. | 2017 | 1111,67 | 1,00 | 4 |
| 73 | Perioperative Goal-Directed Hemodynamic Optimization Using Noninvasive Cardiac Output Monitoring in Major Abdominal Surgery: A Prospective, Randomized, Multicenter, Pragmatic Trial: POEMAS Study (PeriOperative goal-directed thErapy in Major Abdominal Su | 2014 | 553,50 | 12,17 | 4 |
| 74 | The Perioperative Surgical Home: How Anesthesiology Can Collaboratively Achieve and Leverage the Triple Aim in Health Care. | 2014 | 551,33 | 15,67 | 0 |
| 75 | Cell Salvage in Obstetrics. | 2015 | 649,20 | 5,80 | 2 |
| 76 | Repeated Measures Designs and Analysis of Longitudinal Data: If at First You Do Not Succeed-Try, Try Again. | 2018 | 1603,50 | 5,50 | 4 |
| 77 | Brain Monitoring and the Depth of Anesthesia: Another Goldilocks Dilemma. | 2018 | 1603,00 | 3,00 | 10 |
| 78 | Neuraxial Anesthesia in Parturients with Low Platelet Counts. | 2016 | 800,75 | 3,75 | 6 |
| 79 | Best Practices for Postoperative Brain Health: Recommendations From the Fifth International Perioperative Neurotoxicity Working Group. | 2018 | 1600,50 | 9,50 | 12 |
| 80 | Continuous Ultrasound-Guided Adductor Canal Block for Total Knee Arthroplasty: A Randomized, Double-Blind Trial. | 2014 | 530,17 | 11,00 | 11 |
| 81 | Pharmacokinetics and Pharmacodynamics of Drugs Commonly Used in Pregnancy and Parturition. | 2016 | 787,00 | 3,75 | 7 |
| 82 | Impact of Enhanced Recovery After Surgery and Opioid-Free Anesthesia on Opioid Prescriptions at Discharge From the Hospital: A Historical-Prospective Study. | 2017 | 1047,67 | 15,67 | 16 |
| 83 | The Coagulation Profile of End-Stage Liver Disease and Considerations for Intraoperative Management. | 2018 | 1554,50 | 6,00 | 23 |
| 84 | Game Changers: The 20 Most Important Anesthesia Articles Ever Published. | 2015 | 619,60 | 0,60 | 6 |
| 85 | The Incidence and Risk Factors for Perioperative Cardiac Arrest Observed in the National Anesthesia Clinical Outcomes Registry. | 2015 | 616,40 | 8,80 | 5 |
| 86 | Intravenous Ketamine Infusions for Neuropathic Pain Management: A Promising Therapy in Need of Optimization. | 2017 | 1024,67 | 9,00 | 15 |
| 87 | The Use of Postpartum Hemorrhage Protocols in United States Academic Obstetric Anesthesia Units. | 2014 | 505,50 | 4,50 | 1 |
| 88 | The Technology of Processed Electroencephalogram Monitoring Devices for Assessment of Depth of Anesthesia. | 2018 | 1515,50 | 8,00 | 3 |
| 89 | What's New in Obstetric Anesthesia? The 2013 Gerard W. Ostheimer Lecture. | 2014 | 502,67 | 0,33 | 0 |
| 90 | Whole Blood for Resuscitation in Adult Civilian Trauma in 2017: A Narrative Review. | 2018 | 1504,50 | 5,00 | 9 |
| 91 | Opioids for Acute Pain Management in Patients With Obstructive Sleep Apnea: A Systematic Review. | 2018 | 1482,00 | 3,00 | 5 |
| 92 | The Effectiveness of Cricoid Pressure for Occluding the Esophageal Entrance in Anesthetized and Paralyzed Patients: An Experimental and Observational Glidescope Study. | 2014 | 493,33 | 5,67 | 27 |
| 93 | Perioperative Aspirin Management After POISE-2: Some Answers, but Questions Remain. | 2015 | 589,60 | 3,00 | 2 |
| 94 | Postpartum Spinal Cord, Root, Plexus and Peripheral Nerve Injuries Involving the Lower Extremities: A Practical Approach. | 2015 | 587,20 | 1,20 | 1 |
| 95 | The Nuts and Bolts of Performing Focused Cardiovascular Ultrasound (FoCUS). | 2017 | 978,67 | 3,67 | 2 |
| 96 | Writing Research Reports. | 2018 | 1465,50 | 1,00 | 40 |
| 97 | The Effect of Intrathecal Morphine Dose on Outcomes After Elective Cesarean Delivery: A Meta-Analysis. | 2016 | 732,00 | 7,00 | 4 |
| 98 | The Impact of Prehospital Tranexamic Acid on Blood Coagulation in Trauma Patients. | 2018 | 1447,00 | 3,00 | 12 |
| 99 | Nitrous Oxide for Labor Pain: Is It a Laughing Matter?. | 2014 | 480,00 | 1,33 | 2 |
| 100 | Topical and Peripheral Ketamine as an Analgesic. | 2014 | 478,17 | 5,00 | 10 |
